# Serological Cross-Reactivity in Zoonotic Flaviviral Infections of Medical Importance

**DOI:** 10.3390/antib12010018

**Published:** 2023-02-24

**Authors:** Priscilla Gomes da Silva, José Augusto Seixas dos Reis, Marcio Nogueira Rodrigues, Quézia da Silva Ardaya, João Rodrigo Mesquita

**Affiliations:** 1 ICBAS—School of Medicine and Biomedical Sciences, Porto University, 4050-313 Porto, Portugal; 2 Epidemiology Research Unit (EPIunit), Institute of Public Health, Porto University, 4050-600 Porto, Portugal; 3 Laboratório para a Investigação Integrativa e Translacional em Saúde Populacional (ITR), 4050-600 Porto, Portugal; 4 Centro Universitário Fametro, Manaus 69050-000, Brazil

**Keywords:** flaviviruses, original antigenic sin, neutralizing antibodies, cross-reactive antibodies, serological cross-reactivity

## Abstract

Flaviviruses are enveloped RNA viruses from the family Flaviviridae that comprise many important human pathogenic arboviruses such as Yellow Fever, Dengue, and Zika viruses. Because they belong to the same genus, these viruses show sequence and structural homology among them, which results in serological cross-reactivity. Upon infection, the immune system produces both species-specific and cross-reactive antibodies, and depending on the virus, in a successive flavivirus infection, cross-reactive antibodies either enhance protection or exacerbate the disease—the latter usually due to antibody-dependent enhancement. These antigenic relationships between different flaviviruses that lead to serological cross-reactivity make them difficult to be identified through serological methods, especially when it comes to successive flavivirus infections. We present here an overview of the main structural, epidemiological, and immunological aspects of flaviviruses, highlighting the role of neutralizing antibodies in fighting viral infections and in the “original antigenic sin” problem. Finally, we draw attention to the importance of developing a rapid serological diagnostic test for flaviviruses with high sensitivity and specificity, especially when considering that cross-reactive immunity can influence the outcome of these infections.

## 1. Introduction

Flaviviruses are enveloped RNA viruses from the family Flaviviridae, comprising many important human pathogenic arboviruses, including Yellow Fever (YFV), Dengue (DENV), Zika (ZIKV), West Nile (WNV), and Tick-Borne Encephalitis (TBEV) viruses [1]. Because of their widespread and sometimes overlapping distribution, these viruses pose a major threat to global health [2], as demonstrated by the global spread of Dengue, with an estimated 390 million annual infections, the explosive Zika virus epidemics across the Pacific, South and Central America since 2013, and the inherent danger of urban yellow fever in Africa and South America [3,4].

There are two main types of viruses in the Flaviviridae family that can cause infection in mammals: tick-transmitted and mosquito-transmitted viruses [5]. Flaviviruses that are relevant to human disease were organized into eight serocomplexes; an additional seventeen independent viruses appeared not to be antigenically similar enough to be included in a serocomplex (Table 1) [6]. These serocomplexes are defined by the ability of a polyclonal post-immune serum against one flavivirus to neutralize others [6,7].

Because they belong to the same genus, these viruses show sequence and structural homology among them, which sometimes results in serological cross-reactivity of neutralizing antibodies [8]. Upon infection, the immune system produces both species-specific and flavivirus cross-reactive antibodies [2], and depending on the virus, in the case of a successive flavivirus infection, cross-reactive antibodies either enhance protection, as in the case of YFV infection in DENV-immune humans [2], or increase the disease severity, usually due to antibody-dependent enhancement (ADE), as in the case of a second DENV infection [9] or ZIKV infection in DENV-immune humans [10,11,12,13].

One of the most studied examples of ADE regards successive DENV infections [14]. A primary DENV infection induces the production of efficient neutralizing antibodies that coat the virus, but if a second DENV infection of a different serotype occurs, the antibodies produced during the first infection can recognize and bind the second DENV infecting strain but will not be able to properly neutralize it [15,16]. Another extensively studied example concerns ZIKV infection in patients who had been previously infected with DENV, in which neutralizing antibodies for DENV can bind ZIKV virus particles but do not neutralize them efficiently, which will result in a high viral load and ADE of the ZIKV infection [17,18].

These antigenic relationships between different flaviviruses that lead to serological cross-reactivity make them difficult to be diagnosed through serological methods [19,20], especially when it comes to repeated flavivirus infection. The more we understand about immunological cross-reactivity between different flaviviruses, the more precise the diagnosis of these diseases will be.

## 2. Epidemiology and Geographic Distribution of Medically Important Flaviviruses

Projections from the United Nations indicate a likely increase in the global human population up to 9.6 billion by 2050 [21]. Such a population increase would likely favor the spread and impact of zoonoses, as an increasing population density can facilitate the transmission of viruses either directly or through vectors such as mosquitos and ticks. The increased international movement of people through migration, tourism or business travels would also increase the likelihood of a more extensive dissemination of infectious diseases [22]. Moreover, increased land transformation and the disruption of historical ecological processes would promote more contact between humans and infected wildlife or sylvatic vectors [22]. Finally, an increased human population would also increase the formation of breeding sites and habitats for viral vectors such as Aedes aegypti, Aedes albopictus, and Ixodes spp., especially in expanding urban environments [22].

Since 2015, DENV, WNV, and JEV have been responsible for most of the reported flavivirus infections worldwide [23,24]. On average, less than 10% of flavivirus infections are thought to result in clinical symptoms, with complications in an even smaller percentage of cases. If patients develop life-threatening syndromes such as hemorrhagic syndrome or neurological syndrome, the case fatality rate may be as high as 30%; an exception is YFV, as up to 50% of the reported infected people develop clinical symptoms [25,26,27]. The JEV group presents febrile illness and neurological syndromes as its main clinical syndromes. The DENV group presents febrile illness, arthralgia, and in severe cases, shock or hemorrhagic fever. Within the tick-borne flaviviruses, the Asian-Middle East viruses (e.g., Alkhurma virus) are known to cause hemorrhagic syndromes, while the European and American viruses (e.g., tick-borne encephalitis) cause neurological syndromes [25,28,29,30,31,32].

Most known flaviviruses are transmitted horizontally between hematophagous arthropods and vertebrate hosts and are therefore considered dual-host viruses [33]. Flavivirus members are readily grouped into distinct clusters, namely, mosquito-borne, tick-borne, and a group of non-vectored or no-known vector viruses [22]. Not all flaviviruses life cycles alternates between arthropods and vertebrates; some have a vertebrate-specific host range, while others appear to be insect-specific [33].

Flaviviruses have a worldwide distribution, but individual species are restricted to specific endemic or epidemic areas. For example, YFV prevails in tropical and subtropical regions of Africa and South America, DENV in tropical areas of Asia, Oceania, Africa, and the Americas, and JEV in Southeast Asia. In the last 50 years, many flaviviruses, such as DENV, WNV, and YFV, have exhibited great increases in incidence, disease severity, and/or geographic range [34]. Table 2 shows the geographic distribution of human flaviviruses of medical importance, as well as information about their vector and host species.

## 3. Structure of Flaviviruses

**Figure 1:**
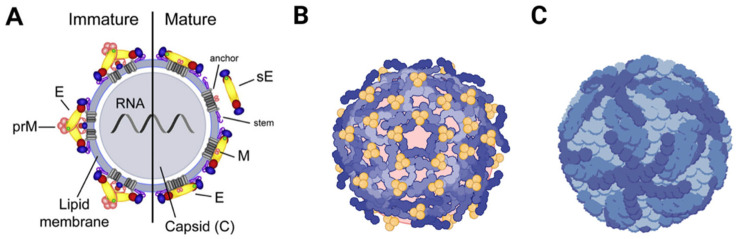
Representation of the mature and immature virion structure of flaviviruses. The surface proteins are arranged in an icosahedral-like symmetry. Mature virions contain two virus-encoded membrane proteins (M and E), while immature virions contain a membrane protein precursor (prM). (**A**) Schematic representation of a mature/immature flavivirus. (**B**) Arrangement representation of E dimers on the surface of an immature DENV virus. (**C**) Arrangement representation of E dimers on the surface of a mature DENV virus.

Regarding their shape, flaviviruses have an icosahedral symmetry and present a spherical envelope around the viral capsid [36]. The capsids are approximately 40–50 nm in diameter and contain only one type of capsid protein, protein C [20]. Mature virions contain proteins M and E, while immature virions contain prM [37] (Figure 1).

PrM is formed by protease hydrolyzation in late viral infection, takes part in the formation of the viral envelope, and plays an important role in determining the E protein’s spatial structure [38]. The E protein monomer is organized into three different envelope domains: I, II, and III (DI, DII, and DIII) [38]. During infection, this protein is responsible for receptor binding and subsequent fusion of the viral membrane with endosomal membranes during endocytosis. All this happens in a low-pH environment that causes the E dimer to dissociate and make the highly conserved fusion peptide exposed at the tip of DII rearrange its domains and form a hairpin-like structure, which is then converted into a trimer [20].

Because of its vital importance in virus entry, the E protein is the major target of flavivirus neutralizing antibodies [20], with the DIII domain being the main antigenic domain because it takes part in crucial events, such as viral attachment, fusion, penetration, hemagglutination, host range, and cell tropism [20,38,39].

The genome of flaviviruses consists of a single open reading region flanked by 5′ and 3′ untranslated regions, both of which with secondary structures essential for the initiation of translation and replication [40]. The translation of the genome by the host cell machinery leads to a single polyprotein—this is a protein expression strategy that results in the generation of many proteins from a single polyprotein precursor through proteolytic cleavages [1].

**Figure 2:**
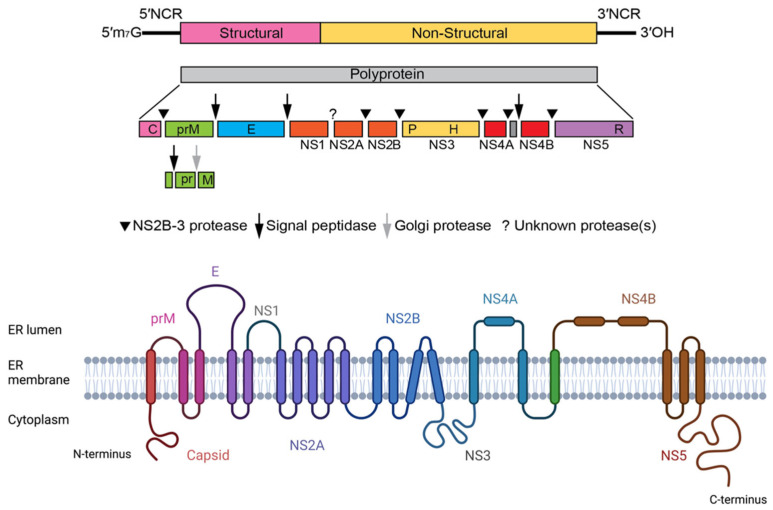
Flavivirus genome structure and chain topology of the translated single polyprotein of ZIKV.

From this polyprotein, three structural proteins can derive, i.e., proteins C (capsid), E (envelope), and prM (precursor of membrane), which, together, compose the viral particle. The rest of the genome encodes the non-structural proteins NS1, NS2A, NS2B, NS3, NS4A, 2K, NS4B, and NS5, which are essential for viral replication [41] (Figure 2).

## 4. Flavivirus Replication Cycle

Flaviviruses enter their host cells through receptor-mediated endocytosis, involving the binding of E glycoproteins to cell surface entry receptors [42,43]. The internalization of the attached virion is mediated by clathrin-dependent endocytosis [43,44]. Once the virion is internalized, the acidic environment of the endosome triggers an irreversible trimerization of the E protein that results in the fusion of the viral and cell membranes [42]. After fusion has occurred, the nucleocapsid is released into the cytoplasm. After penetration of the nucleocapsid into the cytoplasm, the single ORF is translated from the viral RNA, which is disassociated from C proteins, and a precursor polyprotein is co- and post-translationally cleaved by viral and host-encoded proteases [43]. The viral protein involved in the polyprotein processing step is NS3 [45], which possesses helicase, RNA triphosphatase, and serine protease activities [46,47]. The viral ssRNA(+) also serves as a template for the synthesis of new copies of genomic RNA, in which negative-sense RNA is first generated and in turn directs the amplification of new positive-sense RNAs. This viral RNA synthesis is catalyzed by NS5 and its RNA-dependent RNA polymerase (RdRp) activity. During the viral RNA synthesis process, the N-terminal portion of NS5, which has been reported to contain a guanylyltransferase (GTPase) and a methyltransferase (MTase), is involved in the formation of a type 1 cap (m7GpppAmp) structure at the 5′ end of the viral RNA [46,48].

**Figure 3:**
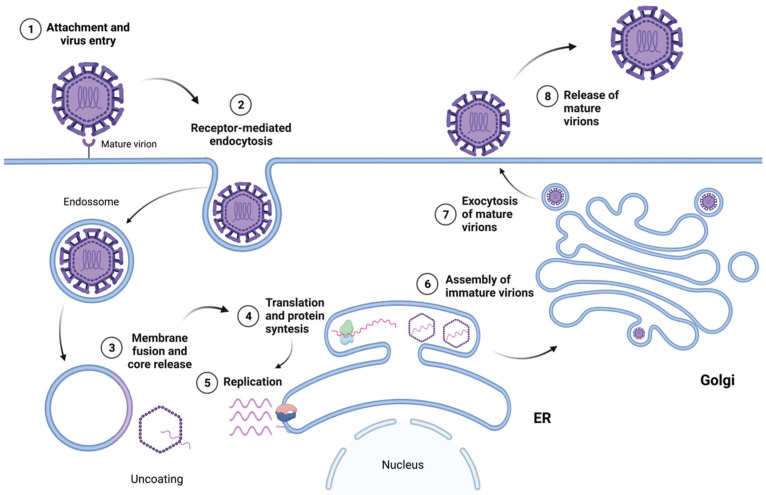
Overview of the flavivirus life cycle.

The nonstructural proteins assemble into the replication complex and drive the invagination of the ER membrane to produce replication organelles. The replication complexes replicate the viral RNA through a negative-strand RNA intermediate, resulting in a positive-strand RNA that is then packaged into new nucleocapsids and envelopes, creating immature virions [45]. Immature virions contain E and prM proteins, a lipid membrane, and the nucleocapsid. The protein prM makes the immature particles non-infectious by repressing the fusogenic activity of the E protein; therefore, immature virions are not able to induce host–cell fusion. These immature virions are secreted in vesicles and enter the trans-Golgi network (TGN), where they progress through chambers. The acidic pH in the TGN causes a rearrangement of the envelope proteins and the proteolytic cleavage of prM into pr and M by the cellular protease furin [49], resulting in mature, infectious particles that then undergo exocytosis [45]. Figure 3 presents an overview of the flavivirus life cycle.

## 5. Immune Response against Viruses and the Importance of Neutralizing Antibodies

The immune response against viruses in humans consists in both a cellular and a humoral immune response [50], and its initiation occurs when the host’s innate immune system recognizes the presence of the virus through Pattern Recognition Receptors (PRRs) that detect Pathogen-Associated Molecular Patterns (PAMPs), such as nucleic acid motifs, viral ssRNA, and dsRNA, and/or Damage-Associated Molecular Patterns (DAMPs), such as reactive oxygen species (ROS), adenosine triphosphate (ATP), or apoptotic/necrotic cells [51,52].

An important cell in the innate immune response during the control of viral infection is the Natural Killer (NK) cell. Besides displaying PRRs that can identify PAMPs or DAMPs, these cells are also sensitive to the level of expression of major histocompatibility I (MHC I) molecules on cell surfaces [52], which are altered in infected cells, thus signaling to the NK cells that they must be destroyed. The way the NK cells do that is through the release of lytic granules stored in their cytoplasm that will act on the target cells and induce apoptosis [50].

After viral recognition by the innate immune system, the antigen-presenting cells (APCs)—usually, dendritic cells (DCs)—are “primed” and proceed to activate the adaptive immune response through the secretion of pro-inflammatory cytokines such as type I interferons (IFNs) and tumor necrosis factor (TNF) [53] and by presenting the antigens to naive T lymphocytes through the major histocompatibility complex II (MHC II), which will result in the activation and clonal expansion of effector and memory B and T cells [54].

Effector T cells include different subsets that are characterized by specialized functions. T helper 1 (Th1) and T helper 2 (Th2) cells are characterized by a strong IFN-γ and IL-4 production, respectively [53], whereas T regulatory cells (Tregs) are characterized by IL-10 and TGF-β production and regulate the immune response by tuning down the effectors’ functions and minimizing immunopathology [53,55]. An efficient antiviral adaptive immune response is considered to be of the Th1 type, but some viruses can inhibit the Th1 response through the downregulation of IFNs production [55].

Once activated, effector T cells (CD4+ cells) will coordinate the adaptive immune response through the production of cytokines, the activation of cytotoxic T cells (CD8+ cells) and effector B cells, and the induction of B cell differentiation into plasma cells, which in turn will proceed to produce specific antibodies to identify, flag, and capture the viral antigens [54].

A subset of effector CD4+ cells known as follicular helper cells (TFH) are involved in B cell differentiation into plasma cells through the release of cytokines and cell–cell interactions, which will then result in the generation of neutralizing antibodies, which are critical components for protection against viruses [53]. Once marked by the antibodies, the pathogens will be recognized and destroyed either by phagocytes or by CD8+ cells.

**Figure 4:**
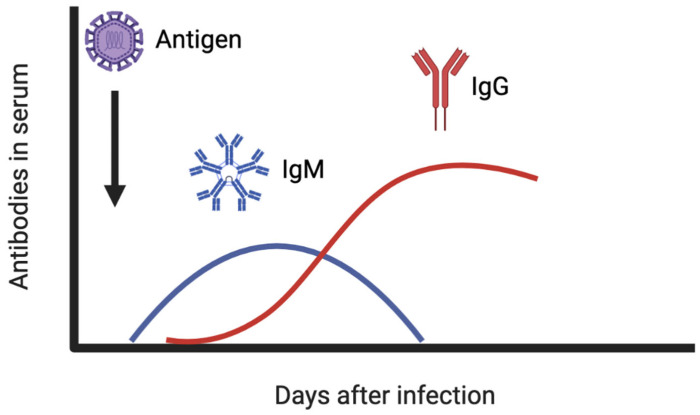
IgM and IgG antibody levels in the serum after viral infection.

At least three classes of immunoglobulins are produced upon virus infection: immunoglobulin G (IgG), IgM, and IgA. Of these three, IgA and IgG are the most important in fighting virus infections. IgA can be found on mucosal surfaces and are essential for the initial protection, whereas IgG directly neutralize viral particles in the serum and other body fluids, being the key immunoglobulin against viral infection [50,56]. IgM commonly arise after 3 days from the infection onset and peak after ~2 weeks, and IgG appear after the first week and maintain high levels in the serum for several months [57]. Figure 4 shows the amount of antibodies present in the serum in the days following an infection.

The neutralizing antibodies prevent the spread of a virus to uninfected cells and allow other defense mechanisms to clean up the infection [50]. Many of the commercial vaccines currently used induce the generation of strong neutralizing antibody responses [53,58], so that in the case of a second exposure to the same pathogen, the immune response will take place in a faster and similar way [54], thus protecting the person from advanced disease development.

There are at least five ways through which neutralizing antibodies can interact with a virus [59]:

1. Steric interference with virus–receptor binding;
2. Blocking of endocytosis;
3. Blocking of the uncoating process;
4. Blocking of the uncoating process inside a cell after replication has started;
5. Aggregation.

**Figure 5:**
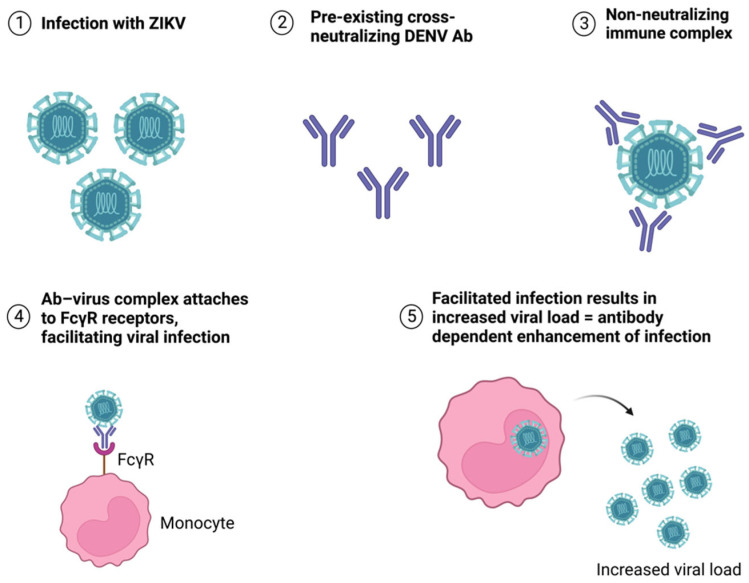
Antibody-dependent enhancement of ZIKV infection caused by pre-existing cross-neutralizing DENV antibodies.

After the infection has been cleared up, non-neutralizing antibodies can also be found in the body. They bind to virus particles but do not interfere with infectivity—the antibody–virus complex can be recognized by the Fc receptors of macrophages, leading to endocytosis of the complex and allowing the viral particles to reproduce inside the cells [56]. As previously mentioned, this is known as antibody-dependent enhancement and is a major problem in the case of secondary infections with a different virus in the same serocomplex [10,12,13,60] (Figure 5).

## 6. The “Original Antigenic Sin” Cross-Reactive Neutralizing Antibodies

The “original antigenic sin” term was first used by Thomas Francis Jr after the observation that “the antibody response to influenza strains from childhood dominates the anti-influenza virus antibody response over time” [61,62], and ever since then the term has been linked to the influenza virus and its variants and presented as the main reason behind the need to create a seasonal influenza vaccine every year.

In a normal immune response to a virus, the human immune system is supposed to identify the viral antigen and activate the innate and adaptive immune responses to fight the intruder [63]. In case of a secondary exposure to the virus, then a secondary stronger, faster, and more efficient response would be expected [63].

What happens in the “original antigenic sin” is that in the case of a secondary exposure to a closely related but still significantly different form of the virus, such as in the case of a secondary DENV infection, the immune response makes a mistake and recognizes this second serotype as the first DENV serotype encountered, which will then result in the production of antibodies specific to the first DENV serotype that would lead to either unsuccessful or delayed clearance of the virus—and, in some cases, can also induce ADE of the disease [54,56].

All flaviviruses are antigenically related and share similar structures [64]. Many of them also have overlapping geographical distribution and are part of the same serocomplex, making the cross-reactions caused by the “original antigenic sin” among these viruses clinically important because, depending on the virus, it can result in cross-protection or exacerbation of the infection [60].

Serological cross-reactivity refers to the ability of antibodies to react to similar antigenic sites on different pathogens, which can have opposite effects: it can result in cross-protection (when the antibodies produced against one virus can enhance protection against another similar virus) or in ADE (when the vector transmission is facilitated, and the severity of the disease is exacerbated) [60].

Flavivirus infections can be diagnosed by virus isolation, detection of virus antigens, viral genome sequencing, and serologic assays [23]. Of all these available diagnostic tests, serological assays are the most widely used in many diagnostics laboratories and hospitals [23]. When it comes to the latter, the are several serological assays that are able to determine the antibody levels against flaviviruses. Among them, there are the Western blotting assay, neutralization tests, hemagglutination–inhibition tests, IgM/IgG antibody-capture ELISAs, and immunofluorescent tests [65]. As infections with flavivirus yield cross-reactive antibodies in addition to species-specific antibodies, there is a concern about the reliability of serological assays for the diagnosis of flaviviruses [52]. This is an important problem for serological diagnosis because the laboratory diagnosis of infection depends on the detection of specific antibodies against the responsible virus [66], and in the presence of cross-reactive antibodies in the serum of the patient, a false-positive result can be obtained, which would result in an inappropriate treatment of the disease [67].

One review that tried to analyze the magnitude of antibody cross-reactivity in medically important mosquito-borne flaviviruses showed that the highest cross-reaction occurred between DENV and non-DENV flaviviruses, especially YFV, and the least cross-reaction was found between CKV virus and DENV [34]. Cross-reaction was also higher on IgG assays than on IgM assays based on the E protein when compared to the NS1protein. In another review, the neutralization test was recommended as the gold standard for the correct diagnosis of flavivirus infection, with DENV serotypes showing the least cross-reactivity by the plaque-reduction neutralization assay (PRNT). In another study, the sera of patients with JEV showed cross-reactivity to WNV, DENV, and TBEV in IgM and/or IgG ELISA, but cross-reactivity was not detected in neutralization tests against DENV and TBE, showing that the neutralization tests are important for the correct diagnosis of JEV [68].

Cross-reactive immunity among DENV, ZIKV, JEV, YFV, and WNV in both human and mouse models was extensively reviewed in a recent paper [69]. In this review, it was highlighted that in human models, pre-existing immunity to DENV had a protective effect against ZIKV infection in newborns; pre-existing immunity to YFV had no effect on the clinical symptoms of a subsequent DENV infection; pre-existing immunity to JEV resulted in an increase in the probability of a symptomatic DENV infection; and lastly, pre-existing immunity to DENV resulted in a significantly better outcome of disease severity after JEV infection.

These findings imply that the reliability of serological test results in areas where more than one flavivirus exists is questionable, with a growing need for the development of diagnostic reagents for flavivirus infections that avoid cross-reactive epitopes to improve the specificity of the serological diagnostic tests [69].

Notwithstanding, the knowledge about cross-protection resulting from neutralizing antibodies can be used to manufacture vaccines [49], implying a clinically significant property of serological cross-reactions. Intra-species cross-reactivity between distinct subtypes is well illustrated by the Encepur (MS) and the FSME-Immun (Pfizer) vaccines for TBE (two different vaccines for TBEV from two different manufacturers) that contain the TBEV-Eu inactivated virus and present cross-reactivity with and cross-protection for TBEV-Fe and TBEV-Sib, based on serological data [70].

When it comes to serological cross-reactivity to flaviviruses, the most extensively studied antibody cross-reactions are the ones between DENV and ZIKV and among all four DENV serotypes. DENV and ZIKV present over 50% sequence homology for their E (envelope) protein [71], whereas the four DENV serotypes have a sequence variation from 30 to 35% [9,12].

ZIKV- and DENV2-immune sera in mice enhanced disease severity after DENV2 infection, while inactivated-DENV vaccination enhanced infection and viremia after ZIKV infection [13]. Another study suggests that in individuals who were previously infected with DENV, a subsequent ZIKV infection would trigger the production of non-neutralizing antibodies or T cell responses specifically directed to DENV, resulting in inefficacy to control the ZIKV infection [54].

Infection with one of the four DENV serotypes does not confer protection against another DENV serotype infection. ADE has been widely studied for this virus and results from the high sequence divergence among DENV serotypes; therefore, the antibodies produced in the first infection do not have enough avidity to neutralize a secondary infection [3,9,10]. However, a cross-reactive response against DENV is not always detrimental for fighting against a secondary infection, as this depends on the variant epitopes encountered in the secondary DENV infection, as shown in an experiment that revealed a possible protective role for IFN levels that correlated with the presence of serotype-specific neutralizing antibodies during the acute phase of the disease [72].

It was also shown that a primary infection with DENV1 or JEV confers protection against a secondary YFV infection in hamsters, and DENV-immune humans present weaker symptoms when infected with YFV [2,73,74].

Currently, there are no licensed vaccines for WNV and ZIKV; however, there are vaccines available against YFV, TBEV, and JEV. In 2019, a single vaccine against DENV from Sanofi Pasteur was licensed under the name of Dengvaxia, but it is indicated only for people who live in endemic areas and who had a laboratory-confirmed previous Dengue infection [75]. There are also vaccines available for YFV, TBEV, and JEV. Challenges such as multiple viral serotypes, incomplete cross-protection, viral interference, and immunological interference (among others) are still a problem in the design of vaccines [76] for these other human pathogenic flaviviruses, reinforcing the need for a better understanding of the adaptive humoral response patterns of neutralizing antibodies against these viruses.

## 7. Conclusions

A clear understanding of serological cross-reactivity patterns among flaviviruses and the consequences of such reactions is important for the correct interpretation of serological tests for the diagnosis of infections and to allow for a correct selection of treatment or other management options, as well as for the design of new vaccines against flaviviruses, as no commercial vaccine has been approved yet.

Currently, there is an urgent need for the development of a rapid serological diagnostic test that has high sensitivity and specificity, especially when considering that cross-reactive immunity influences the outcome of flavivirus infections. Further studies are needed in order to identify a single antigen for serological diagnostic tests that can provide high sensitivity and specificity, as this is the main setback when it comes to the diagnosis of flavivirus infections through serological methods.

## Figures and Tables

**Table 1:** Flaviviruses classification into serocomplexes.

Antigenic Complex (Serocomplex)	Viruses
Tick-borne encephalitis	Russian spring-summer encephalitis
	Central European encephalitis
	Omsk hemorrhagic fever
	Louping Ill
	Kyasanur Forest disease
	Langat
	Phnom Penh bat
	Carey Island
	Negishi
	Powassan
	Karshi
	Royal Farm
Rio Bravo	Rio Bravo
	Entebbe bat
	Dakar bat
	Bukalasa bat
	Saboya
	Apoi
Japanese encephalitis	Japanese encephalitis
	Murray Valley encephalitis
	Kokobera
	Alfuy
	Stratford
	St. Louis encephalitis
	Usutu
	West Nile
	Kunjin
	Koutango
Tyuleniy	Tyuleniy, Saumarez
	Reef
	Meaban
Ntaya	Ntaya
	Temusu
	Yokose
	Israel turkey meningoencephalitis
	Bagaza
Uganda S	Uganda S
	Banzi
	Bouboui
	Edge Hill
Dengue	Dengue 1
	Dengue 2
	Dengue 3
	Dengue 4
Modoc virus complex	Modoc
	Cowbone Ridge
	Jutiapa Sal
	Vieja
	San Perlita

**Table 2:** Main ecological characteristics and geographic distribution of human pathogenic flaviviruses. Adapted from [35].

Virus	Country Where It Was Discovered	Geographic Distribution	Main Vector spp.	Main Host spp.	Human Disease
Alkhurma	Saudi Arabia	Arabian Peninsula ^a^	*Ornithodorus* *savignyi* ^a^	Humans, sheep, camels	Haemorrhagic fever
Apoi	Japan	Japan	Unknown	Rodents ^a^	Encephalitis
Bagaza	Central African Republic	Africa	*Culex* spp.	Unknown	Fever
Banzi	South Africa	Africa	*Culex* spp.	Unknown	Fever
Bussuquara	Brazil	Brazil	*Culex* spp.	Unknown	Fever
Dakar bat	Senegal	Africa	Unknown	Bats ^a^	Fever
Dengue 1	Hawaii	Tropics, subtropics	*Aedes aegypti*	Humans	Fever, rash, vasculopathy
Dengue 2	New Guinea	Tropics, subtropics	*Aedes aegypti*	Humans	Fever, rash, vasculopathy
Dengue 3	Philippines	Tropics, subtropics	*Aedes aegypti*	Humans	Fever, rash, vasculopathy
Dengue 4	Philippines	Tropics, subtropics	*Aedes aegypti*	Humans	Fever, rash, vasculopathy
Ilheus	Brazil	South and Central America	*Culex* spp. ^a^	Birds	Fever
Japanese encephalitis	Japan	Asia and Oceania	*Culex trita-eniorhynchus*	Birds	Encephalitis
Koutango	Senegal	Senegal	Unknown	Rodents ^a^	Fever, rash
Kyasanur Forest disease	India	India	*Haemaphysalis* *spinigera*	Monkeys	Haemorrhagic fever
Langat	Malaysia	Malaysia, Thailand, Siberia	*Ixodes granulatus*	Unknown	Encephalitis
Louping ill	Scotland	UK, Ireland; it has also been reported in Norway and one region in far eastern Russia, and on the island of Bornholm in Denmark	*Ixodes* spp.	Sheep, grouse, hares	Encephalitis
Modoc	USA	USA	Unknown	*Peromyscus maniculatus*	Encephalitis
Murrat Valley encephalitis	Australia	Australia, New Guinea	*Culex annulirostris*	Birds	Encephalitis
Ntaya	Uganda	Africa	Mosquitos	Unknown	Fever
Omsk haemorrhagic fever	Russia	Western Siberia	*Dermacentor pictus*	Muskrats, rodents ^a^	Haemorrhagic fever
Powassan	Russia, USA, Canada	Russia, USA, Canada	*Ixodes* spp.	Small mammals	Encephalitis
Rio Bravo	USA	USA, Mexico	Unknown	*Tadanida braziliensis* *mexicana*	Fever
Rocio	Brazil	Brazil	*Culex* spp. ^a^	Birds	Encephalitis
St Louis encephalitis	USA	North, Central, and South America	*Culex* spp.	Birds	Encephalitis
Sepik	New Guinea	New Guinea	Mosquitos	Unknown	Fever
Spondweni	South Africa	Africa	*Aedes* *circumluteolus*	Unknown	Fever
Tick-borne encephalitis	Russia	Many parts of Europe and Asia	*Ixodes* spp.	Rodents ^a^	Encephalitis
Usutu	South Africa	Africa	Mosquitoes	Birds	Fever, rash
Wesselsbron	South Africa	Parts of Africa, Madagascar, and Thailand	*Aedes* spp.	Unknown	Unknown
West Nile	Uganda	Worldwide	Mosquitos, ticks	Birds	Encephalitis
Yellow Fever	Ghana	Tropical and subtropical areas of Africa and South America	*Aedes* *spp/Haemagogus* spp.	Monkeys	Pantropic
Zika	Uganda	Africa, the Americas, Southern Asia and Western Pacific	*Aedes* spp.	Monkeys ^a^	Fever, rash

^a^ The principal host identified is probably correct.
